# Two Case Reports of Isolated Intramuscular Cysticercosis: An Uncommon Pediatric Pseudotumor

**DOI:** 10.7759/cureus.68206

**Published:** 2024-08-30

**Authors:** Shubhi Gaur, Pratap S Parihar, Devyansh Nimodia

**Affiliations:** 1 Department of Radiodiagnosis, Jawaharlal Nehru Medical College, Datta Meghe Institute of Higher Education and Research, Wardha, IND

**Keywords:** mri, albendazole, swelling, children, tapeworm

## Abstract

*Taenia solium*, the pig tapeworm, produces larvae that cause cysticercosis, a common parasitic disease of the human nervous system including the brain. The disease is native to countries like Central and South America, Eastern Europe, Africa, India, and Indonesia. Cysticercosis is endemic in North India, particularly in Bihar, Uttar Pradesh, and Punjab. Asymptomatic cysts may have a history of trauma, while lower extremity involvement is less common. Isolated muscle involvement typically has no lethal consequences. Two cases, both pediatric, were diagnosed with intramuscular cysticercosis without involvement of the brain parenchyma. The patients received oral prednisolone therapy for seven days, followed by albendazole for 28 days. The swellings decreased in size and no new swellings or symptoms appeared during the two- and four-week follow-ups. At a three-month follow-up, the swellings completely resolved.

Neurocysticercosis is a commonly encountered infection of the human central nervous system and one of the major causes of acquired epilepsy globally. Most cases are asymptomatic and go undiagnosed, with the first case likely due to trauma. Diagnosis is often delayed or overlooked due to vague clinical symptoms. Clinical differential diagnoses for intramuscular cysticercosis include lipomas, epidermoid cysts, neuromas, neurofibromas, pseudoganglia, sarcomas, myxomas, pyomyositis, cold abscess, and tuberculous lymphadenitis. High-resolution ultrasound is the most accurate method for diagnosing intramuscular cysticercosis, as it is quick, simple, and less expensive. Muscular cysticercosis sonographic patterns can be categorized into four types: first degree, uneven, irregular, and calcified. Magnetic resonance imaging (MRI) is the most accurate way to diagnose intramuscular cysticercosis, as it can show live scolex, cysts, and degenerating cysts. In every case, there is edema to varied degrees, with fluid-filled lesions without peripheral enhancement visible in early stages and peripheral rim augmentation and perilesional edema observed in later stages.

## Introduction

The parasitic disease cysticercosis is brought on by the ingested larvae of *Taenia solium* also called the the pig tapeworm [1]. It is the most common human nervous system parasite infection in immunocompetent people affecting primarily the brain and occurs all over the world [2]. However, isolated intramuscular involvement of cysticercosis is a rare occurrence with only a few case reports available [3,4]. Although it is native to places like Western countries of Central and South America, Eastern Europe, Africa, and eastern countries of India, China, and Indonesia, it has now become sporadic as a result of increased travel and immigration [5]. In India, cysticercosis seems to be widely endemic in North India, especially in the states of Bihar, Uttar Pradesh, and Punjab [6].

Children frequently suffer due to increased fomite infection risk; however, a population of any age or gender may catch this infection with a history of migration across endemic regions in the past [3,7]. Vegetarians and nonvegetarians may both contract the disease [8]. The clinical features of this disease depend on the burden of infection, the affected location, and the inflammation that accompanies the inflammatory process [3,9].

Usually, there is no fever. A history of trauma or acute infection could have caused inflammation in cysts which had been lying dormant, owing to the host's immune response at the time. Asymptomatic patients and those exhibiting painful, sensitive swellings at numerous places are both possible clinical presentations. Lower extremity involvement has less frequently been observed than head, neck, trunk, and upper extremity involvement; however, the reason for this prevalence in the head is unknown [10,11]. Isolated muscle involvement, in contrast to neurocysticercosis, typically has no lethal consequences [12]. Three different clinical presentations for the muscular form have been identified: the masslike type known as myalgic type, the abscess like type which mimics a pseudotumor, and the uncommon pseudohypertrophic type [13].

The radiological investigations used for intramuscular cysticercosis include ultrasonography (USG) and magnetic resonance imaging (MRI) with USG being the primary investigation and MRI being the gold standard; however, both these investigations usually act as an adjunct to each other. USG is helpful in identifying the characteristic appearances of the hypoechoic cysts which may or may not show hyperechogenic scolex, while MRI provides helpful insights in staging of the disease and demonstrating perilesional edema. Here, we present two cases, both pediatric, presenting with inflammatory swellings of the extremities, who underwent radiological investigations and were diagnosed as cases of intramuscular cysticercosis, without involvement of the brain parenchyma.

## Case presentation

Case 1

**Figure 1:**
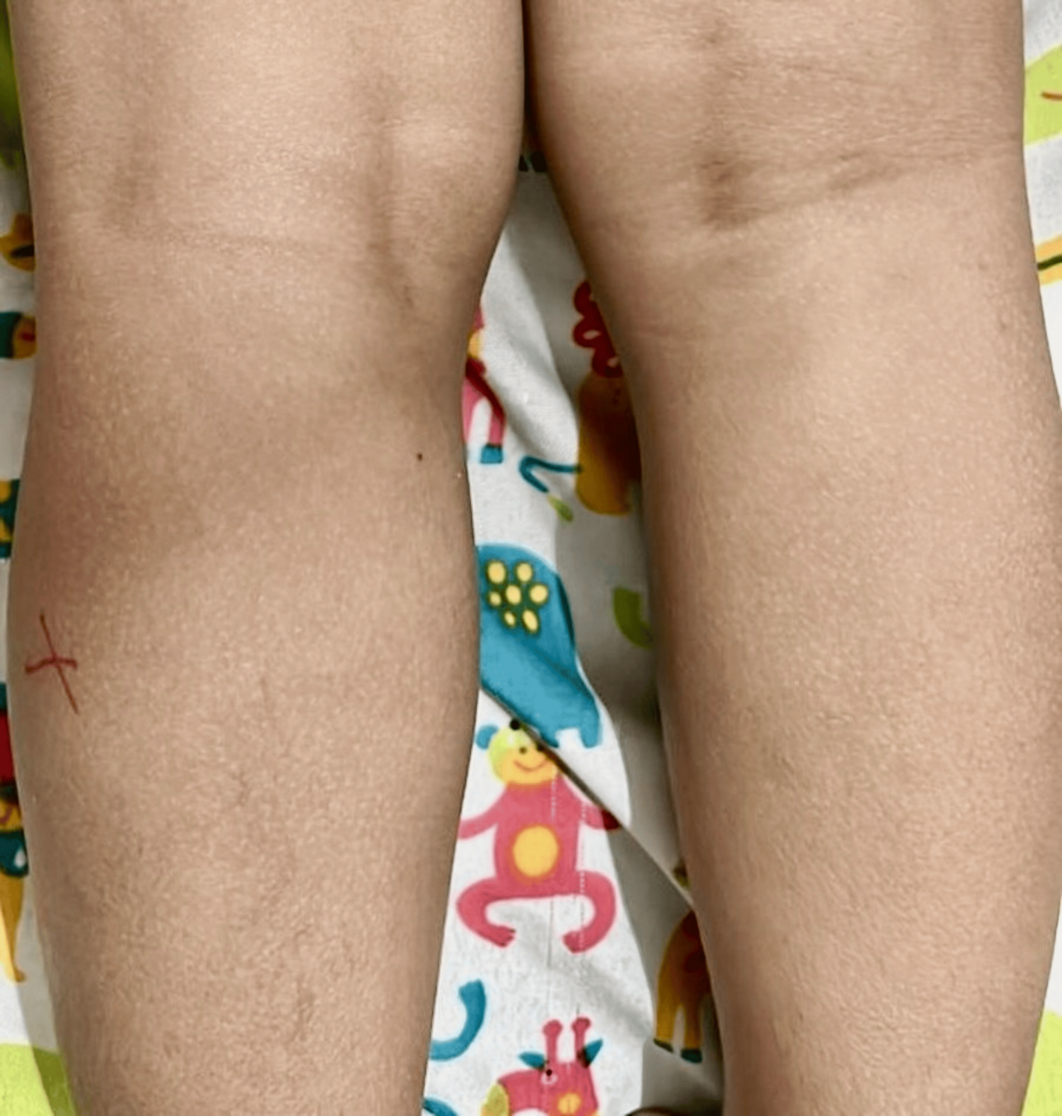
Clinical image of the left knee showing a well-defined swelling (marked by red X over the skin), without overlying skin changes

An 18-month-old boy presented with a lump over the posterior aspect of the left knee joint which was noticeable for two months. On examination, the lump was not painful, did not reduce with pressure, and was not fixed. The overlying skin did not show any signs of inflammation (Figure 1). The lump was diffuse, ill-defined, and firm. Clinically, the dimensions measured were 3.5 × 2.5 cm. On probing further, the patient did not give any history of fever, cough, or past infectious history of tuberculosis in the family or elsewhere. There was no history of meat or poultry intake as the child was herbivorous. General physical examination including pulse rate and blood pressure was normal. The complete blood counts and other investigations did not reveal any abnormality.

**Figure 2:**
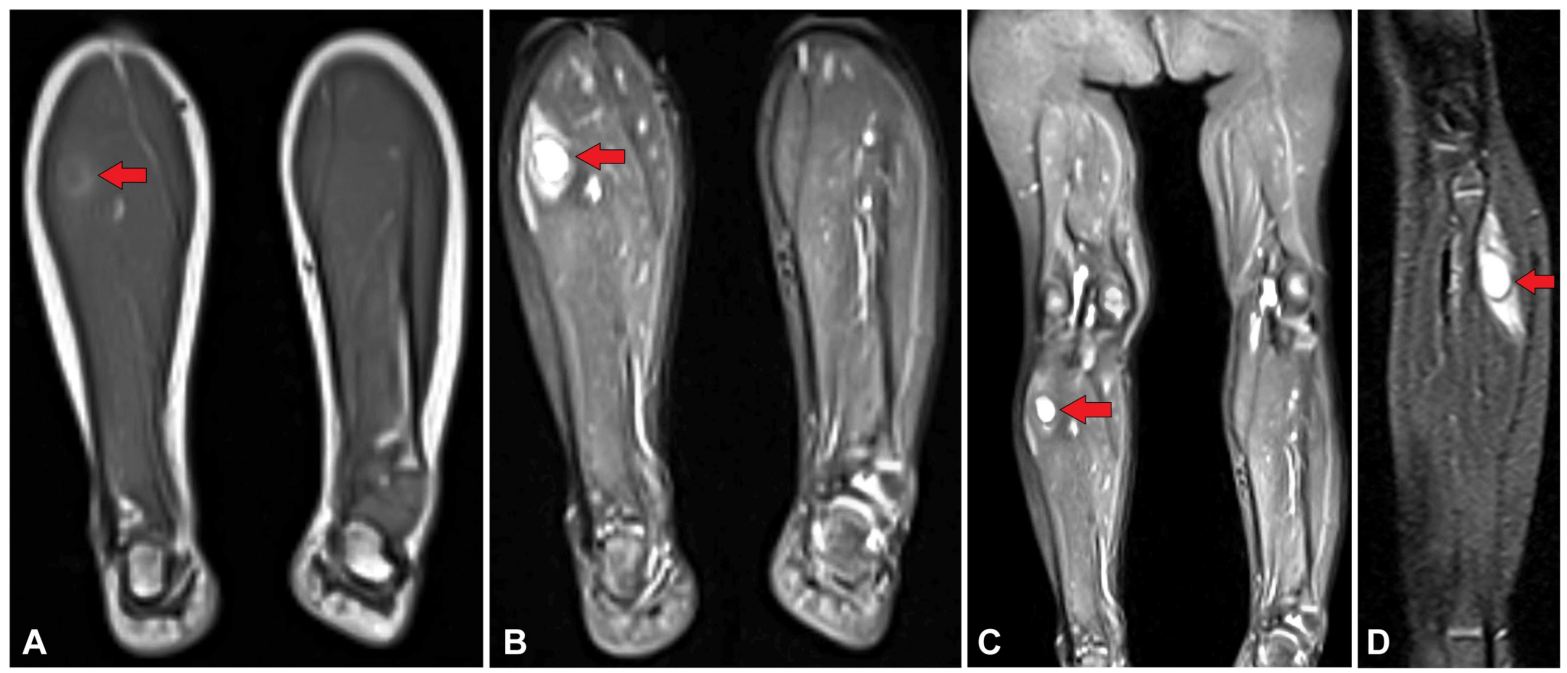
MRI images (in prone position) showing altered signal intensity lesion in the posterosuperior aspect of calf muscles of the left leg appearing (A) hypointense on T1WI (B), (C) hyperintense on T2WI, and (D) hyperintense on STIR sagittal image with perilesional edema (red arrow) MRI: Magnetic resonance imaging; WI: weighted image; STIR: short tau inversion recovery

MRI was done further which showed altered signal intensity lesion in the superior aspect of left calf muscles involving myofascial planes appearing hyperintense on short tau inversion recovery (STIR) sequence and isointense on T1-weighted image (WI) measuring approximately 2 x 1.9 x 2 cm with surrounding perilesional edema (Figure 2).

Case 2

**Figure 3:**
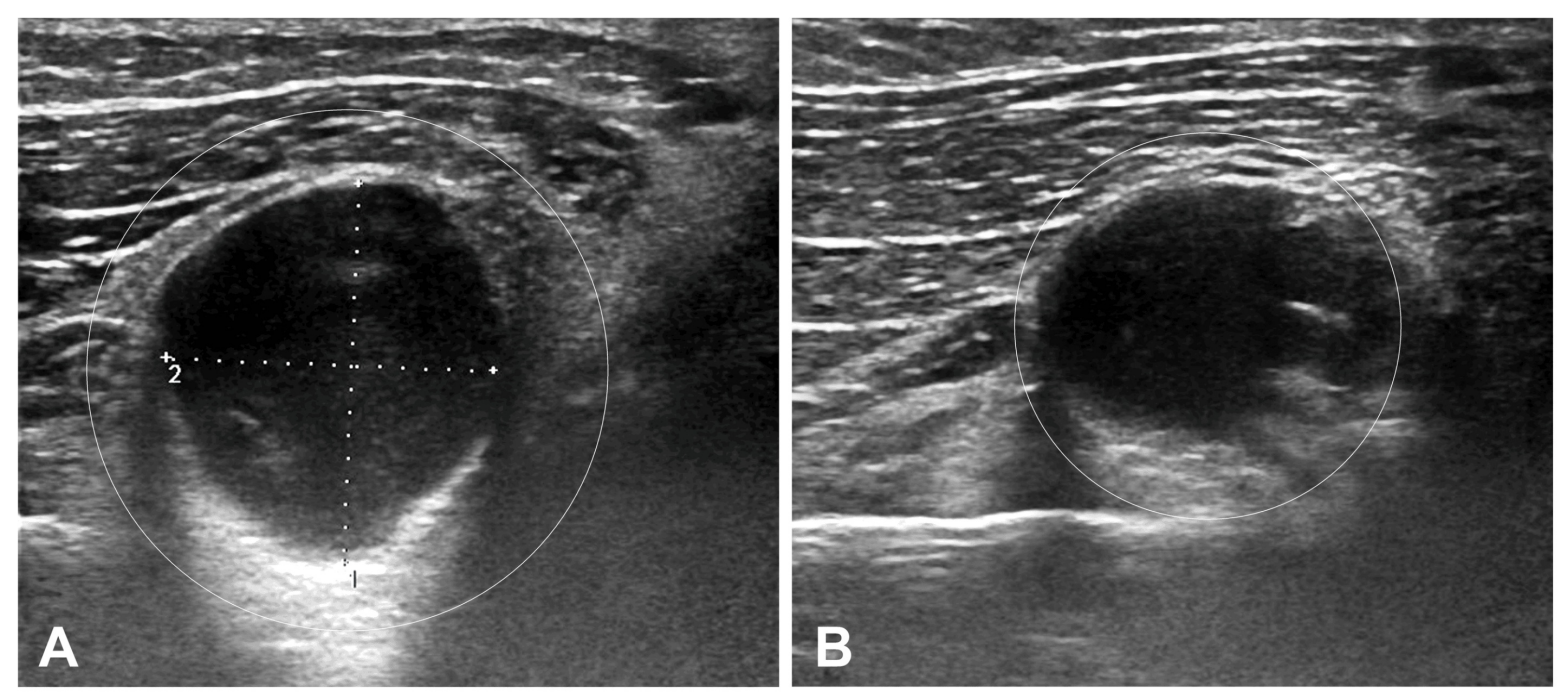
Grayscale B mode ultrasound image showing a well-defined, round-to-oval, hypoechoic lesion in the intramuscular plane of the left forearm giving posterior acoustic enhancement in (A) transverse and (B) sagittal planes. There is no evidence of internal septations, echoes, or solid component within the cystic lesion

Another case is that of a 17-year-old female who came with complaints of painless swelling over the ventral aspect of arm since two months. On inspection, the swelling measured to that a size of lemon, and there was no evidence of any discharging sinus or local inflammation over the overlying skin. On palpation, the swelling was nontender, firm, and nonslippable. The patient had no complaints pertaining to the nervous system. USG of the local site revealed well-defined cystic swelling showing posterior acoustic enhancement, measuring 18 x 15 mm, and taking no vascularity on Doppler (Figure 3).

**Figure 4:**
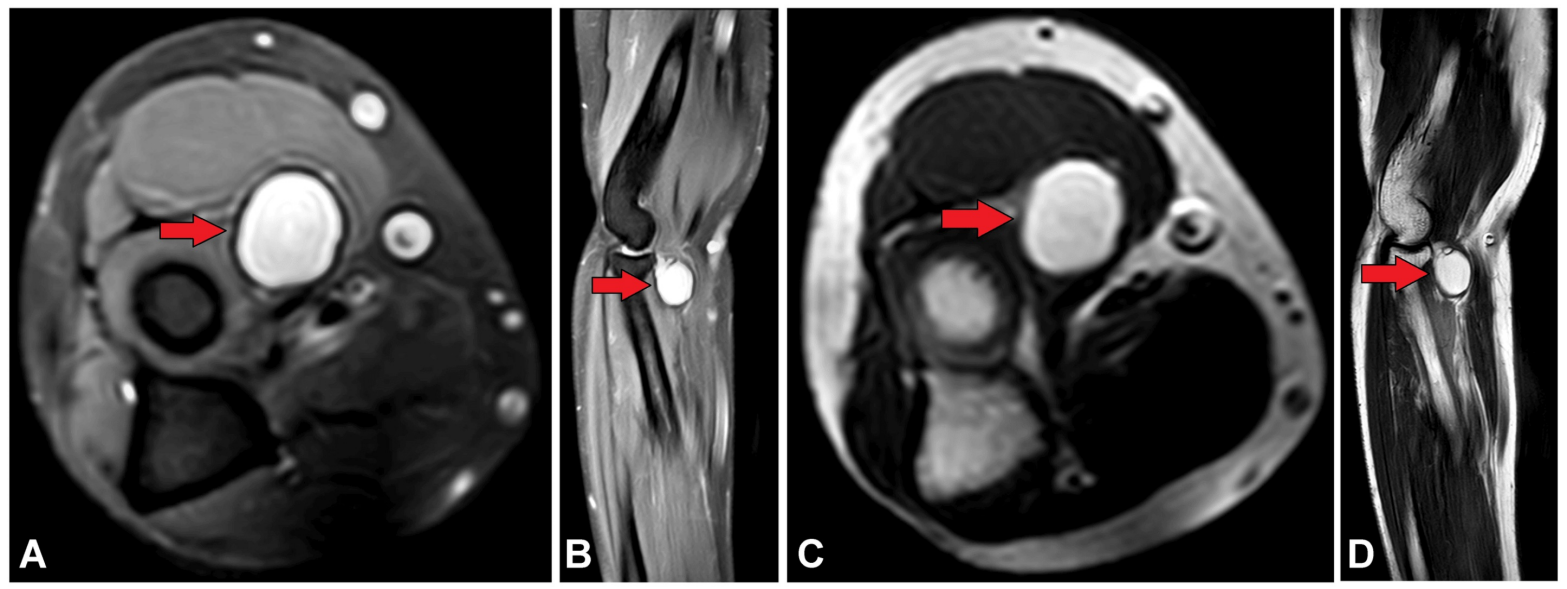
MRI images of the arm showing a well-defined altered signal intensity lesion in the intramuscular plane of brachioradialis muscle in the cubital fossa just anterior to the radial head, appearing hyperintense (red arrow) on T2WI in (A) axial section, (B) sagittal section, and hyperintense on T1WI in (C) axial plane and (D) and sagittal plane MRI: Magnetic resonance imaging

She was further investigated for an MRI elbow which showed a well-defined cystic altered signal intensity lesion noted intramuscularly in the brachioradialis muscle in the cubital fossa just anterior to the radial head, appearing isointense on T1WI, hyperintense on T2WI and STIR and showing no blooming on susceptibility weighted imaging (SWI) (Figure 4).

For seven days, the patients received oral prednisolone therapy. After the first three days of steroid treatment, albendazole (15 mg/kg/day) was started and administered for 28 days. The swellings had decreased in size and no new swellings or symptoms had appeared during the two- and four-week follow-ups. At a three-month follow-up, the swellings had completely clinically resolved.

## Discussion

A commonly acquired infection of the central nervous system and a frequent cause of acquired epilepsy globally is neurocysticercosis. Most of the time, the central nervous system and muscles are engaged [14]. It is uncommon for a single cyst to exclusively affect one muscle [12]. For the patient's entire life, the majority of muscle cysticercosis is asymptomatic and goes undiagnosed. Rarely, the release of antigens from a cyst following worm death or cyst trauma causes an immunological response and inflammation surrounding the cyst, which makes the cyst symptomatic [3].

In the first instance, our primary clinical differential diagnosis was either lipoma or neurofibroma. It seemed quite unlikely that pyomyositis and tuberculous lymphadenitis would develop in the absence of constitutional symptoms. Trauma was likely the source of the first case in this study, which led to antigen release and subsequent inflammation. Initial impressions in case two included tenosynovitis and tuberculous myositis.

Clinical differential diagnoses for intramuscular cysticercosis include lipomas, epidermoid cysts, neuromas, neurofibromas, pseudoganglia, sarcomas, myxomas, pyomyositis, cold abscess, and tuberculous lymphadenitis [13,15]. Due to vague clinical symptoms, intramuscular cysticercosis diagnosis is frequently delayed or overlooked [15]. Thus, a radiological evaluation is frequently required to make an early diagnosis.

The most accurate way to diagnose intramuscular cysticercosis is by MRI. When determining the disease's stage and displaying perilesional edema, MRI is preferable to CT scanning. Live scolex, cysts, and degenerating cysts can all be seen on an MRI. The cyst is usually elliptical or oval in shape and oriented along the muscle fibers; on T1WI, it is hypointense, while on T2WI, it is hyperintense. Post-contrast perilesional enhancement is present [14]. In every case, there is edema to varied degrees. When the parasite is alive and in the early stages, a fluid-filled lesion without peripheral enhancement is visible. The patient may or may not have symptoms at this point. Peripheral rim augmentation and perilesional edema are observed in the later stage, which is caused by fluid leakage and the host reaction that follows [16].

Ultrasound has the benefits of being a quick, inexpensive, noninvasive modality without exposure to harmful radiation. It is easy to complete and simple to repeat with little to no discomfort for the patient. The intramuscular cyst has the recognizable look of an eccentric echogenic scolex on ultrasound [13,14]. Muscular cysticercosis sonographic patterns have been characterized in four different ways [16].

The first form, caused by the demise of the larva, is a cysticercus cyst with an inflammatory mass surrounding it. The second kind is an irregular cystic lesion which has less liquid content on one side, which might suggest fluid extravasation. This cyst lacks the ectopic echogenic protrusion from the wall caused by the scolex. This kind of look could result from scolex escaping the cyst or the cyst partially collapsing [16].

In the third form, a significant irregular accumulation of infective fluid which is intramusclular and a cysticercus cyst containing the scolex. This can be caused by a persistent inflammatory response around the cyst. An intramuscular abscess may resemble this condition in appearance. The cysticercus form itself, appearing as an oval or round well-defined cystic lesion with an eccentric echogenic scolex in it, is the diagnostic feature in all three of these sorts of presentations. The fourth type of cyst is calcified, and it shows up as many elliptical calcifications in soft tissue [16].

Upon comparing the literature available on similar cases [3,4], it was devised that the first case which showed intense perilesional edema on MRI belonged to the second stage, while the second case which did not show any perilesional edema or scolex presented in the inflammatory first stage. Based on the MRI and ultrasound characteristic features, other differentials such as lipoma which suppresses on STIR and epidermoid cyst which does not usually give confluent perilesional edema were ruled out. Subsequently, USG and MRI are used for follow-up after treatment and can pick up any residual disease with similar findings or help track progress of the disease into its final calcified stage wherein the calcified cysts will give posterior acoustic shadowing on USG and SWI in MRI will show blooming in magnitude phase and corresponding hyperintense signal in phase image.

## Conclusions

One should always consider isolated myocysticercosis as a differential diagnosis when a patient from an endemic zone presents with a tiny muscle pseudotumor that has an unclear etiology. In tiny cysts, serum indicators have little sensitivity and the blood picture may be deceptive. MRI and USG are undoubtedly noninvasive methods of diagnosing and staging this condition which can help the clinician to opt for adequate treatment. With the help of a combination of oral antihelminthic and steroid preparation, this cyst can be fully treated without surgery. However, if the symptoms persist or if surgical removal of the cyst is indicated, cystectomy of the lesion can be a helpful method.
